# Digital eye strain among Indian university students in the post-COVID era: a cross-sectional study

**DOI:** 10.3389/fpubh.2026.1829471

**Published:** 2026-06-08

**Authors:** Swati Kumari, Yamini Michelle Maran, Kshitij A. Krishnan, Mahalakshmi Raja, Gayatri S., Vinishaa Paraman

**Affiliations:** 1 Department of Microbiology, Sri Ramachandra Institute of Higher Education and Research, Chennai, India; 2 Department of Ophthalmology, Sri Ramachandra Institute of Higher Education and Research, Chennai, India

**Keywords:** computer vision syndrome, digital eye strain, digital health, post COVID-19, screen time, SDG 3

## Abstract

**Background:**

Digital Eye Strain (DES), also known as Computer Vision Syndrome (CVS), has become increasingly prevalent with the expansion of digital device use, particularly in academic settings. University students represent a high-risk group due to prolonged screen exposure. This study aimed to estimate the prevalence of DES using a validated instrument and to examine its association with screen time and related factors.

**Methodology:**

A cross-sectional analytical study was conducted among undergraduate students in Chennai, India, between January and March 2025. A structured questionnaire, including the validated Computer Vision Syndrome Questionnaire (CVS-Q), was administered using a non-probability convenience sampling approach through institutional academic groups. DES was defined as a CVS-Q score ≥6. Screen time was analyzed across all exposure categories. Descriptive statistics, non-parametric tests, and logistic regression analyses were performed.

**Results:**

Of all undergraduate students invited to participate in the study, 1,070 responded to the questionnaire. After excluding 24 incomplete responses, 1,046 participants were included in the final analysis (mean age 20 years; 61.8% female). Overall, 94.1% reported at least one symptom, and 73.7% met the criteria for DES (CVS-Q ≥6). Median CVS-Q scores increased progressively with screen time (*P* < 0.001). Logistic regression analysis demonstrated a significant association between increasing screen time and DES (*P* < 0.001). Female participants had significantly higher symptom scores (*P* = 0.003). Increased screen time was also associated with higher odds of headache, with the highest risk observed in the >10 h group (OR = 6.53; 95% CI: 2.14–19.92; *P* = 0.001).

**Conclusion:**

DES is highly prevalent among undergraduate students and is associated with prolonged screen exposure and modifiable ergonomic factors, highlighting the need for structured preventive strategies, including digital hygiene awareness and ergonomic education within academic institutions, while representing a modifiable, lifestyle-associated health condition that aligns with SDG 3: Good Health and Wellbeing (Target 3.4), emphasizing prevention and promotion of wellbeing.

## Introduction

Digital Eye Strain (DES), also referred to as Computer Vision Syndrome, is a growing digital health concern associated with prolonged use of visual display devices. It describes a constellation of ocular and extraocular symptoms resulting from sustained screen exposure, including headache, eye strain, blurred vision, dryness, burning sensation, and musculoskeletal discomfort involving the neck and shoulders (1).

With the global expansion of digital technology, particularly in academic environments, DES has emerged as an important public health concern. University students represent a particularly vulnerable population due to sustained near-work activity, high smartphone dependency, and increasing reliance on digital platforms for learning and communication (2, 3).

The transition to online and hybrid education during the COVID-19 pandemic further intensified digital exposure, contributing to a rise in visual and postural complaints among young adults (4).

Prolonged digital device use has been identified as a key risk factor for DES, with increasing duration of screen exposure associated with greater symptom burden. Environmental and ergonomic factors such as glare, improper viewing distance, poor posture, and inadequate screen breaks further contribute to symptom development (5, 6).

Epidemiological studies have reported a wide range of DES prevalence across student populations, largely due to differences in diagnostic criteria and assessment tools. Meta-analyses estimate a pooled global prevalence of approximately 66%−69%, whereas studies in India have reported high prevalence among university students (7, 8). However, variability in methodology and lack of standardized diagnostic instruments limit comparability across studies (7).

Post-pandemic studies among university students have reported very high DES prevalence, in some cases exceeding 90%, likely reflecting variations in case definitions and exposure patterns. Environmental and ergonomic factors such as glare, prolonged near work, and poor workstation setup further contribute to symptom development, while extended use of visual display terminals and shorter viewing distances increase the risk of headache and eyestrain (5, 9, 10).

The Computer Vision Syndrome Questionnaire (CVS-Q), developed by Seguí et al. (11) is a standardized and validated instrument for diagnosing DES using a structured frequency–intensity scoring system with a defined cutoff (score ≥6). The use of such validated tools enables more reliable and comparable epidemiological estimates and supports the development of institutional digital health policies (12). Despite increasing digital dependency in Indian higher education, awareness and systematic evaluation using validated instruments remain limited (13). Moreover, few studies in Indian university populations have examined dose–response relationships between screen time and symptom severity using multivariable models that account for ergonomic and behavioral factors (8). Addressing these gaps is essential to generate context-specific evidence to inform preventive strategies within academic settings.

In this context, the present study aimed to estimate the prevalence of DES using the validated CVS-Q (cutoff ≥6), examine the association between daily screen time and DES severity, and identify independent demographic and ergonomic predictors.

As a modifiable, lifestyle-associated condition affecting visual and mental wellbeing, DES aligns with SDG 3: Good Health and Wellbeing (Target 3.4), which emphasizes prevention and reduction of non-communicable disease burden. We hypothesized that longer daily screen exposure would be associated with higher DES scores in a dose-dependent manner; furthermore, we hypothesized that factors such as gender, posture, viewing distance, and suboptimal ergonomic practices would independently predict increased DES severity.

## Materials and methods

### Study design and setting

This cross-sectional analytical study was conducted among undergraduate students enrolled at a university in Chennai, India, between January and March 2025. The study aimed to estimate the prevalence of DES and evaluate its association with daily screen exposure and selected ergonomic factors in an academic setting. The study was designed and reported in accordance with the Strengthening the Reporting of Observational Studies in Epidemiology (STROBE) guidelines.

### Participants and sampling

Undergraduate students aged 18–35 years who reported using digital devices, including smartphones, laptops, tablets, or computers, were eligible for inclusion. Students with a history of diagnosed ocular pathology (e.g., glaucoma, cataract), recent ocular surgery, or active ocular infection at the time of the study were excluded.

A non-probability convenience sampling approach was employed. Participants were primarily recruited during scheduled classroom sessions across different undergraduate programs.

At the end of lecture sessions across various university streams, the study authors visited the lecture hall and provided a brief, standardized oral explanation of the study objectives, the voluntary nature of participation, and confidentiality. Following this, the link to a structured questionnaire hosted on Google Forms was shared with students through their class-specific WhatsApp groups. Subsequently, a short briefing on digital hygiene practices was conducted.

Students were given adequate time to complete the questionnaire prior to dispersal. This hybrid recruitment strategy facilitated the inclusion of students from the MBBS (four academic years; approximately 250 students per batch), Allied Health Sciences, and Engineering streams.

As a non-probability convenience sampling approach with voluntary participation was employed, the possibility of selection bias cannot be entirely excluded.

All eligible participants were included, and daily screen time was analyzed as an exposure variable across predefined categories.

### Sample size estimation

The sample size was calculated assuming a 95% confidence level and a 3% margin of error. Based on these parameters, the minimum required sample size was 1,067 participants. To account for potential non-response and incomplete questionnaires, the target sample size was increased to 1,100 participants. The target sample was proportionally distributed across academic streams, including MBBS (*n* = 750), Allied Health Sciences (*n* = 190), and Engineering (*n* = 160), based on institutional enrolment data.

### Data collection procedure

Data were collected using a structured, self-administered questionnaire administered via Google Forms. Participation was voluntary, and informed consent was obtained electronically prior to data collection. Responses were collected anonymously.

Responses were restricted to one submission per participant using Google Forms settings (single-response restriction) to minimize duplicate entries. A brief standardized orientation was provided prior to survey completion to ensure informed participation.

### Outcome and exposure measures

#### Digital eye strain assessment

Digital Eye Strain was assessed using the Computer Vision Syndrome Questionnaire (CVS-Q), a validated instrument developed by Seguí et al. (11). The CVS-Q evaluates 16 symptoms based on frequency and intensity, and scores are calculated using a standardized algorithm. A total score ≥6 was used to define clinically significant DES. The instrument has demonstrated good reliability and validity in previous studies.

#### Exposure variable

The primary exposure variable was daily screen time, assessed both as a continuous and a categorical variable. Screen time was categorized as < 2, 2–6, 6–10, and >10 h. Additional exposure variables included the type of digital device used, duration of use, and frequency of device usage per week.

#### Covariates

Potential confounding variables included age, gender, academic program, use of corrective lenses, and ergonomic practices such as posture, viewing distance, screen height, lighting conditions, presence of glare, and awareness of preventive measures, including the 20-20-20 rule.

### Statistical analysis

Data were entered and analyzed using Stata version 17.0 (Stata Corp LLC, College Station, TX, USA). Descriptive statistics were used to summarize participant characteristics and estimate the prevalence of DES. Continuous variables were expressed as mean ± standard deviation or median with interquartile range, as appropriate. Categorical variables were presented as frequencies and percentages.

The distribution of CVS-Q scores was assessed and found to be non-normal (positively skewed). Therefore, non-parametric tests were used for analysis. The Mann–Whitney *U*-test was used for comparisons between two groups, and the Kruskal–Wallis test was used for comparisons across multiple groups. A *P*-value ≤ 0.05 was considered statistically significant.

Logistic regression analysis was performed with DES (defined as CVS-Q score ≥6) as the primary dependent variable. Daily screen time was the main exposure variable, and demographic and ergonomic factors—including age, gender, academic program, use of corrective lenses, posture, viewing distance, screen height, lighting conditions, glare, and awareness of the 20-20-20 rule—were included as covariates.

Adjusted odds ratios (aORs), 95% confidence intervals (CIs), and *P*-values were reported. Headache was analyzed as a secondary symptom-specific outcome, as it was the most frequently reported symptom.

Given the multiple statistical comparisons performed in this study, there is an increased risk of type I error (false-positive findings). No formal adjustment for multiple testing was applied; therefore, the results, particularly from secondary analyses, should be interpreted with caution.

### Ethical considerations

The study was approved by the Institutional Ethics Committee (CSP-III/24/JUL/08/313) and conducted in accordance with the Declaration of Helsinki. Participation was voluntary, and informed consent was obtained from all participants. Data were collected anonymously, and confidentiality was maintained throughout the study.

## Results

### Participant flow and characteristics

Of all undergraduate students invited to participate in the study, 1,070 responded to the questionnaire. After excluding 24 incomplete responses, the final analytical sample consisted of 1,046 participants.

The participants had a mean age of 20 years, with 61.8% female participants. Regarding digital device use, 93.8% (*n* = 981) reported using digital devices for more than 2 h daily, and 92.4% (*n* = 966) primarily used smartphones (Table 1).

**Table 1:** Sociodemographic characteristics and screen usage patterns (*n* = 1,046).

Variable	*n*	%
Total no of participants	1,046	100
Male	400	38.20
Female	646	61.80
Age group (years)		
< 18	89	8.5
18–20	854	81.6
>20	103	9.85
Daily screen time		
< 2 h	65	6.2
2–6 h	551	52.7
6–10 h	358	34.2
>10 h	72	6.9

### Prevalence of digital eye strain

The prevalence of DES was high in this study. Overall, 94.1% (*n* = 984) of participants reported at least one symptom, while 73.7% (*n* = 771) met the criteria for clinically significant DES (CVS-Q score ≥6).

### Symptom profile

**Figure 1:**
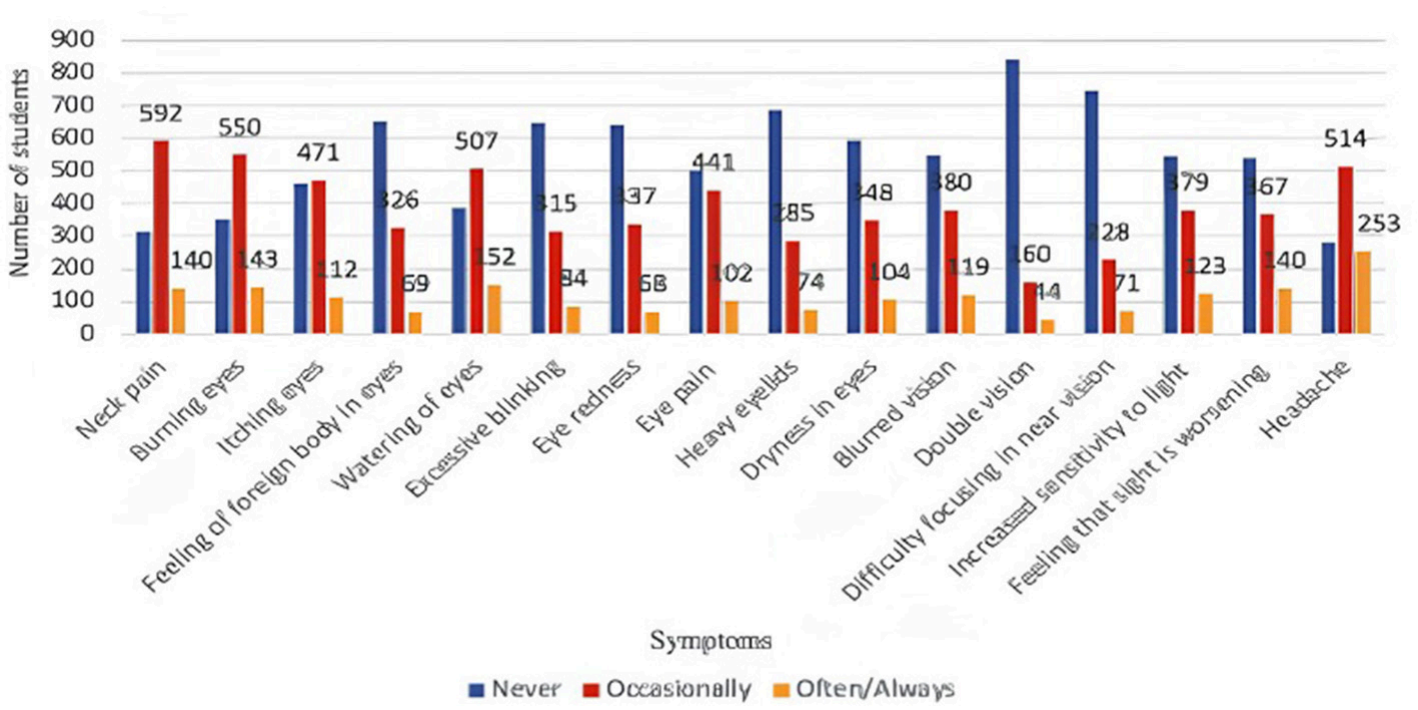
Distribution of computer vision syndrome symptoms among undergraduate students. Frequency of reported ocular and extraocular symptoms categorized as “Never,” “Occasionally,” and “Often/Always.”

The most commonly reported symptom was headache (73.3%, *n* = 767), followed by neck and shoulder pain (69.9%, *n* = 702) and burning sensation in the eyes (66.2%, *n* = 693). The least reported symptom was diplopia (19.5%, *n* = 204; Figure 1).

### Gender differences

A Mann–Whitney *U*-test demonstrated a statistically significant difference in CVS-Q scores between male and female participants (*P* = 0.003), with higher scores observed among female students.

### Association between screen time and DES severity

Median CVS-Q scores increased progressively with increasing daily screen time. A Kruskal–Wallis test demonstrated a statistically significant difference across screen time categories (*P* < 0.001; Table 2), indicating a dose–response relationship between screen exposure and symptom severity. The median CVS-Q scores across different screen time categories, showing a progressive increase with longer exposure (< 2 h: 5; 2–6 h: 9; 6–10 h: 11; >10 h: 14.5). The median score of 5 in the < 2 h group lies below the diagnostic cutoff of 6, indicating that this group, on average, does not meet the threshold for DES, which is consistent.

**Table 2:** Association between daily screen time and CVS-Q scores.

Screen time category	Median CVS-Q score	Interquartile range (IQR)	*P*-value
< 2 h	5	(3–7)	
2–6 h	9	(6–13)	
6–10 h	11	(8–16)	
>10 h	14.5	(10–20)	< 0.001^*^

*P*-value obtained using the Kruskal–Wallis test. ^*^means statistical significant.

Binary logistic regression analysis was conducted to examine the association between screen time and the presence of Digital Eye Strain (DES), using < 2 h of screen exposure as the reference category (Table 3). The analysis revealed a strong and statistically significant dose–response relationship between increasing screen time and DES. Participants with 2–6 h of screen time had significantly higher odds of DES (OR: 28.3; 95% CI: 11.2–71.1; *P* < 0.001), while those with 6–10 h demonstrated even greater odds (OR: 72.3; 95% CI: 28.0–186.6; *P* < 0.001). Individuals reporting >10 h of screen exposure exhibited markedly elevated odds of DES (OR: 852.0; 95% CI: 100.4–7,226.5; *P* < 0.001). These findings indicate a robust and graded increase in the likelihood of Digital Eye Strain with prolonged screen use, highlighting a clear exposure–response relationship.

**Table 3:** Analysis of the association between screen time and digital eye strain (CVS).

Screen time category	Odds ratio (OR)	95% confidence interval	*P*-value
< 2 h	Reference	—	—
2–6 h	28.3	11.2–71.1	< 0.001
6–10 h	72.3	28.0–186.6	< 0.001
>10 h	852.0	100.4–7,226.5	< 0.001

### Ergonomic and behavioral factors

More than half of the participants (56.9%) reported maintaining a viewing distance of less than 40 cm, while 42.8% were aware of the 20-20-20 rule.

A statistically significant association was observed between seating posture and the number of symptoms (*P* = 0.012). Students with poor posture were 4.64 times more likely to report symptoms than those with proper posture (OR = 4.64; 95% CI: 1.63–13.21; *P* = 0.004).

### Association between screen time and headache

Binary logistic regression analysis was performed to assess the association between daily screen time and the likelihood of reporting headache (Table 4), with the < 2 h group serving as the reference category. Compared with participants reporting < 2 h of screen time per day, those with 2–6 h had higher odds of headache (OR = 2.86; 95% CI: 1.45–5.64; *P* = 0.003), followed by those with 6–10 h (OR = 5.36; 95% CI: 2.56–11.20; *P* < 0.001) and >10 h (OR = 6.53; 95% CI: 2.14–19.92; *P* = 0.001). These findings demonstrate a graded increase in the odds of headache with increasing screen time.

**Table 4:** Logistic regression analysis of the association between daily screen time and headache.

Screen time category	Odds ratio (OR)	95% confidence interval	*P*-value
< 2 h (reference)	1.00	—	—
2–6 h	2.86	1.45–5.64	0.003
6–10 h	5.36	2.56–11.20	< 0.001
>10 h	6.53	2.14–19.92	0.001

## Discussion

DES has emerged as an increasingly recognized consequence of prolonged use of digital devices in academic and occupational settings (1, 14). University students represent a particularly vulnerable population due to sustained near-work activity, high smartphone dependence, and increasing reliance on digital platforms for academic engagement (4, 15). The shift to online and hybrid learning during the COVID-19 pandemic has further increased screen exposure, contributing to a rise in visual and musculoskeletal complaints among young adults (16). Although several international studies have examined the prevalence of DES, variability in diagnostic criteria, study designs, and assessment tools has limited comparability across populations (7). In the Indian context, post-pandemic data using standardized diagnostic instruments remain limited. This study was therefore designed to estimate the prevalence of DES among undergraduate students using the validated Computer Vision Syndrome Questionnaire (CVS-Q) and to examine its association with daily screen exposure.

A key strength of this study is the use of the validated CVS-Q, which enhances reliability and comparability across populations (2, 7). The questionnaire was administered in English, and as participants were enrolled in an English-medium academic curriculum, functional proficiency was assumed. However, no formal cultural adaptation or linguistic validation was performed for Indian undergraduates, and subtle interpretative differences cannot be entirely excluded.

This study found that 94.1% of students reported at least one symptom, and 73.7% met the validated diagnostic threshold for DES. Meta-analyses have estimated the global pooled DES prevalence at 66%−69% (2, 7), whereas pre-pandemic Indian studies have reported prevalence ranging from 77 to 82% (8, 17, 18). In contrast, post-pandemic studies have documented higher rates, including 84.8% in Egypt, 94.5% in Jordan, and 97.3% in Saudi Arabia (19–21), with similar findings reported among Thai university students in virtual classrooms (4). The elevated prevalence observed in this study likely reflects sustained digital academic exposure in the post-pandemic era, consistent with increased screen time during COVID-19 (16). Importantly, while minor intermittent symptoms may be common among digital users, the proportion meeting the validated cutoff (73.7%) indicates a clinically relevant symptom burden.

A notable contribution of this study is the demonstration of a graded association between daily screen exposure and symptom severity. Median CVS scores increased progressively across exposure categories, and logistic regression analysis demonstrated a significant association between higher screen exposure and DES. Similar exposure–response patterns have been reported in occupational and student populations (10, 12, 22). Sustained visual display terminal use has been associated with increased accommodative demand and ocular discomfort, and the graded relationship observed in this study supports the role of cumulative exposure as an important determinant of symptom burden (23).

Headache and neck–shoulder pain were among the most frequently reported symptoms. Prior studies among office workers and students have similarly identified headache and musculoskeletal discomfort as predominant complaints (22, 24, 25). Smartphone-related posture and prolonged cervical flexion have been associated with neck strain (15, 26). Headache was additionally analyzed as it represents both the most prevalent symptom and a clinically relevant manifestation of DES.

Ergonomic studies have demonstrated associations between screen exposure, trapezius muscle activation, and musculoskeletal symptoms (5, 27, 28). Female students in this study demonstrated significantly higher CVS scores, consistent with findings from systematic reviews and university-based studies (7, 21, 29). Experimental studies have suggested differential visual stress responses in females during prolonged computer tasks; however, the present study did not investigate underlying biological mechanisms (5).

This study employed non-probability convenience sampling through class WhatsApp groups, which may limit representativeness and introduce participation bias, particularly if symptomatic individuals were more likely to respond. The high response rate observed in this study may be attributable to in-class dissemination of the survey and immediate completion by participants, which differs from typical online survey response patterns and may reduce non-response bias. However, the results should not be interpreted as population representativeness.

Daily screen time and symptoms were self-reported, introducing potential recall bias and exposure misclassification. Objective digital usage tracking was not performed, and no ophthalmic examination was conducted. Refractive errors, dry eye disease, or other ocular conditions were not clinically confirmed. Prior literature emphasizes the importance of distinguishing symptom-based DES from clinically verified ocular pathology (14, 30).

The cross-sectional design limits causal inference. Although a dose–response association was observed, temporal directionality between exposure and symptom development cannot be definitively established. The multiple statistical comparisons performed in this study may increase the risk of type I error (false-positive findings); therefore, findings, particularly those from secondary analyses, should be interpreted with caution.

Screen time was initially considered as a potential eligibility criterion; however, all respondents were included in the final analysis to enable assessment across the full exposure gradient. Additionally, as this study was conducted within a single medical college in Chennai, the findings may not be fully representative of the broader undergraduate population in Chennai or India.

The findings of this study demonstrate a high burden of DES and highlight it as a modifiable digital health issue within academic settings. Preventive strategies may include structured ergonomic education, institutional digital hygiene campaigns, classroom-based visual reminders for breaks, workstation optimisation, and integration of digital wellness counseling into campus health services. Such interventions are supported by existing occupational and clinical literature (1, 24, 30) and align with Sustainable Development Goal 3 by promoting preventive health strategies targeting modifiable lifestyle risk factors among young adults.

## Conclusion

DES was highly prevalent among undergraduate students in this study, with nearly three-quarters demonstrating clinically significant symptom scores based on the validated CVS-Q. Higher daily screen exposure was associated with greater symptom severity and increased odds of headache, indicating a clear exposure–response relationship.

These findings highlight DES as an important and potentially modifiable digital health concern within academic settings. Ergonomic factors, including posture and viewing distance, were also associated with symptom burden, suggesting opportunities for targeted preventive interventions.

However, these findings should be interpreted in light of certain limitations. Screen time and symptoms were self-reported, which may introduce recall bias and misclassification of exposure. Objective ophthalmic assessments were not performed, and, given the cross-sectional design, causal relationships cannot be inferred; the findings should be interpreted strictly as associations rather than as cause–effect relationships.

Overall, the study underscores the need for structured institutional strategies, including ergonomic education, digital hygiene awareness, and promoting healthy screen-use practices. Future multicentric and longitudinal studies incorporating objective measures of screen exposure and clinical evaluation are warranted to better establish causal pathways and inform evidence-based interventions.

## Data Availability

The raw data supporting the conclusions of this article will be made available by the authors, without undue reservation.
