# Supplementary material for: Digital eye strain among Indian university students in the post-COVID era: a cross-sectional study
Source: Front Public Health. 2026 Jun 8;14:1829471. doi: 10.3389/fpubh.2026.1829471 (PMC13284132; doi:10.3389/fpubh.2026.1829471)
Supplement: Supplementary file 1 [file Table_1.docx]

| **Table 1: Prevalence of Computer Vision Syndrome Among Student Populations Across Various Countries** | | | | | | |
| --- | --- | --- | --- | --- | --- | --- |
| **S.No** | **Author** | **year** | **Country** | **Sample size** | **Prevalence (%)** | **Study subjects** |
|  |  |  |  |  |  |  |
|  |  |  |  |  |  |  |
| 1 | Han CC, et al. [1] | 2013 | China | 1469 | 57 | Students |
| 2 | Reddy, et al. [2] | 2013 | Malaysia | 795 | 89.9 | Students |
| 3 | Logaraj M, et al. [3] | 2014 | Tamil Nadu, India | 215 | 81.8 | Students |
| 4 | Noreen K, et al. [4] | 2016 | Pakistan | 198 | 67.2 | Students |
| 5 | Singh H, et al. [5] | 2016 | Madhya Pradesh, India | 150 | 32.2 | Students |
| 6 | Hassan, et al.[6] | 2016 | Pakistan | 170 | 72.4 | Students |
| 7 | Hashemi H, et al. [7] | 2017 | Iran | 415 | 90.4 | Students |
| 8 | Mansoori N, et al. [8] | 2017 | Pakistan | 150 | 50.8 | Students |
| 9 | Iqbal, et al. [9] | 2018 | Egypt | 100 | 86 | Students |
| 10 | Kharel, et al. [10] | 2018 | Nepal | 236 | 71.6 | Students |
| 11 | Arshad S, et al. [11] | 2019 | Pakistan | 320 | 58.1 | Students |
| 12 | Ranganatha SC, et al. [12] | 2019 | Karnataka, India | 150 | 86.7 | Students |
| 13 | Anupama, et al. [13] | 2019 | Telangana, India | 300 | 60.3 | University students |
| 14 | Patil, et al. [14] | 2019 | Maharashtra, India | 463 | 77.5 | University students |
| 15 | Abudawood GA, et al. [15] | 2020 | Saudi Arabia | 587 | 95.1 | University students |
| 16 | Sawaya R, et al. [16] | 2020 | Beirut | 457 | 77.5 | University students |
| 17 | Al Tawil, et al. [17] | 2020 | Saudi Arabia | 713 | 45.2 | University students |
| 18 | Kumar, et al. [18] | 2020 | Tamil Nadu India | 60 | 85 | University students |
| 19 | Niveditha, et al. [19] | 2020 | Tamil Nadu,  India | 250 | 82.4 | University students |
| 20 | Altalhi, et al. [20] | 2020 | Saudi Arabia | 334 | 97.3 | University students |
| 21 | Cantó-Sancho N, et al. [21] | 2021 | Spain | 244 | 76.6 | University students |
| 22 | Gammoh Y, et al. [22] | 2021 | Jordan | 382 | 94.5 | University students |
| 23 | Noreen K, et al. [23] | 2021 | Pakistan | 326 | 98.7 | University students |
| 24 | Nwankwo B, et al.  [24] | 2021 | Nigeria | 153 | 54.2 | University students |
| 25 | Wang L, et al. [25] | 2021 | China | 300 | 32 | University students |
| 26 | Zenbaba H, et al. [26] | 2021 | Ethiopia | 416 | 70.43 | University students |
| 27 | Turki, et al. [27] | 2021 | Saudi Arabia | 139 | 92.8 | University students |
| 28 | Huyhua, et al. [28] | 2021 | Peru | 119 | 85.7 | University students |
| 29 | Iqbal, et al. [29] | 2021 | Egypt | 4030 | 84.8 | University students |
| 30 | Akowuah, et al. [30] | 2021 | Ghana | 362 | 64.4 | University students |
| 31 | Selvaraj, et al. [31] | 2021 | Tamil Nadu,  India | 253 | 35.2 | University students |
| 32 | Fernandez-Villacorta, et al. [32] | 2021 | Peru | 106 | 62.3 | University students |
| 33 | Iqbal (a), et al. [33] | 2021 | Egypt | 733 | 76 | University students |
| 34 | Wangsan K, et al. [34] | 2022 | Thailand | 527 | 100 | University students |
| 35 | Munsamy, et al. [35] | 2022 | South Africa | 290 | 64.1 | University students |
| 36 | Almousa, et al. [36] | 2022 | Saudi Arabia | 300 | 67 | University students |
| 37 | Wangsan, et al. [37] | 2022 | Thailand | 527 | 81 | University students |
| 38 | Coronel, et al. [38] | 2022 | Paraguay | 228 | 82.5 | University students |
| 39 | Girum, et al. [39] | 2022 | Ethiopia | 812 | 41.7 | University students |
| 40 | Imran, et al. [40] | 2022 | Saudi Arabia | 172 | 66.9 | University students |
| 41 | Uwimana, et al.[41] | 2022 | China | 452 | 50 | University students |
| 42 | Lotfy, et al. [42] | 2022 | Egypt | 412 | 88.8 | University students |
| 43 | Simanta, et al. [43] | 2022 | Bangladesh | 917 | 68.2 | University students |
| 44 | Lindo-Cano, et al. [44] | 2022 | Peru | 709 | 58.3 | University students |
| 45 | Estrada, et al. [45] | 2022 | Peru | 215 | 71.1 | University students |
| 46 | Al-Darrab, et al. [46] | 2022 | Saudi Arabia | 521 | 96 | University Students |
| 47 | Gerena, et al. [47] | 2022 | Colombia | 296 | 85.8 | University Students |
| 48 | Vargas-Rodriguez, et al. [48] | 2023 | Colombia | 300 | 78 | University Students |

**Table 5** presents a compilation of published studies reporting the prevalence of Computer Vision Syndrome (CVS) among students in different countries from 2013 to 2023. It includes information on the year of publication, country, sample size, and reported CVS prevalence rates. The table reflects wide variability in CVS prevalence influenced by factors such as screen time duration, digital learning environments, ergonomic awareness, and regional digital usage trends.

References:

1. Han CC, Liu R, Liu RR, Zhu ZH, Yu RB, Ma L. Prevalence of asthenopia and its risk factors in Chinese college students. International journal of ophthalmology. 2013 Oct 18;6(5):718. doi: 10.3980/j.issn.2222-3959.2013.05.31
2. Reddy, S. C., Low, C., Lim, Y., Low, L., Mardina, F., & Nursaleha, M. (2013). Computer vision syndrome: a study of knowledge and practices in university students. *Nepalese Journal of Ophthalmology*, *5*(2), 161–168. <https://doi.org/10.3126/nepjoph.v5i2.8707>
3. Logaraj M, Madhupriya V, Hegde SK. Computer vision syndrome and associated factors among medical and engineering students in Chennai. Annals of medical and health sciences research. 2014;4(2):179-85. Doi: 10.4103/2141-9248.129028

## Noreen K, Batool Z, Fatima T, Zamir T. Prevalence of computer vision syndrome and its associated risk factors among under graduate medical students of urban karachi. Pakistan Journal of Ophthalmology. 2016 Sep 30;32(3). DOI: <https://doi.org/10.36351/pjo.v32i3.106>

1. Singh S, McGuinness MB, Anderson AJ, Downie LE. Interventions for the Management of Computer Vision Syndrome: A Systematic Review and Meta-analysis. Ophthalmology. 2022 Oct;129(10):1192-1215. doi: 10.1016/j.ophtha.2022.05.009
2. Hassan HM, Ehsan S, Arshad HS. Frequency of computer vision syndrome & ergonomic practices among computer engineering students. Int J Sci Res. 2016;5(5):121-5.doi: [10.3980/j.issn.2222-3959.2013.05.31](https://doi.org/10.3980/j.issn.2222-3959.2013.05.31)
3. Hashemi H, Saatchi M, Yekta A, Ali B, Ostadimoghaddam H, Nabovati P, Aghamirsalim M, Khabazkhoob M. High Prevalence of Asthenopia among a Population of University Students. J Ophthalmic Vis Res. 2019 Oct 24;14(4):474-482. doi: 10.18502/jovr.v14i4.5455.
4. Mansoori N, Qamar N, Mubeen SM. Dry eye syndrome and associated risk factors among computer users in Karachi, Pakistan. ANNALS OF ABBASI SHAHEED HOSPITAL AND KARACHI MEDICAL & DENTAL COLLEGE. 2017 Sep 30;22(3):165-70.DOI:[10.58397/ashkmdc.v22i3.123](http://dx.doi.org/10.58397/ashkmdc.v22i3.123)
5. Iqbal M, Elzembely H, Elmassry A, Elgharieb M, Assaf A, Ibrahim O, Soliman A. Computer vision syndrome prevalence and ocular sequelae among medical students: a university-wide study on a marginalized visual security issue. The Open Ophthalmology Journal. 2021 Sep 22;15(1).DOI:[10.2174/1874364102115010156](http://dx.doi.org/10.2174/1874364102115010156)
6. Sitaula RK, Khatri A. Knowledge, attitude and practice of computer vision syndrome among medical students and its impact on ocular morbidity. doi:http://dx.doi.org/10.3126/jnhrc.v16i3.21426
7. Arshad S, Qureshi MF, Ali M, Piryani K, Shafqat K, Mateen M, Waheed M, Rohaila M, Amin M. Computer vision syndrome: prevalence and predictors among students. Ann Psychophysiol. 2019 Oct 12;6(1):15-22. Doi: 10.29052/2412-3188.v6.i1.2019.15-22
8. Sitaula K, Kafle N, Acharya A, Mishra VP. Prevalence and associated factors of computer vision syndrome among the computer engineering students of Pokhara University affiliated colleges of Kathmandu valley. Int J Community Med Public Health. 2020 Jun;7(6):2027-31.DOI: <http://dx.doi.org/10.18203/2394-6040.ijcmph20202448>
9. Pulla A, Samyuktha N, Kasubagula S, Kataih A, Banoth D, Addagatla H. A cross sectional study to assess the prevalence and associated factors of computer vision syndrome among engineering students of Hyderabad, Telangana. Doi:https://doi.org/10.18203/2394-6040.ijcmph20185264
10. Patil A, Chaudhury S, Srivastava S. Eyeing computer vision syndrome: Awareness, knowledge, and its impact on sleep quality among medical students. Industrial psychiatry journal. 2019 Jan 1;28(1):68-74.DOI: [10.4103/ipj.ipj_93_18](https://doi.org/10.4103/ipj.ipj_93_18)
11. Abudawood GA, Ashi HM, Almarzouki NK. Computer vision syndrome among undergraduate medical students in King Abdulaziz University, Jeddah, Saudi Arabia. Journal of Ophthalmology. 2020;2020(1):2789376. Doi: <https://doi.org/10.1155/2020/2789376>
12. Sawaya RI, El Meski N, Saba JB, Lahoud C, Saab L, Haouili M, Shatila M, Aidibe Z, Musharrafieh U. Asthenopia among university students: the eye of the digital generation. Journal of family medicine and primary care. 2020 Aug 1;9(8):3921-32. doi: [10.4103/jfmpc.jfmpc_340_20](https://doi.org/10.4103/jfmpc.jfmpc_340_20)
13. Al Tawil L, Aldokhayel S, Zeitouni L, Qadoumi T, Hussein S, Ahamed SS. Prevalence of self-reported computer vision syndrome symptoms and its associated factors among university students. *European Journal of Ophthalmology*. 2018;30(1):189-195. doi:[10.1177/1120672118815110](https://doi.org/10.1177/1120672118815110)
14. Kumar BS. A study to evaluate the knowledge regarding computer vision syndrome among medical students. Biomed Pharmacol J. 2020 Mar;13(1):469-73. Doi: <https://dx.doi.org/10.13005/bpj/1907>
15. Niveditha KP, Dheepak Sundar M. Digital vision syndrome (DVS) among medical students during COVID-19 pandemic curfew. Int J Res Pharm Sci. 2020;11(1):1128-33. DOI:[10.26452/ijrps.v11iSPL1.3557](http://dx.doi.org/10.26452/ijrps.v11iSPL1.3557)
16. Altalhi A, Khayyat W, Khojah O, Alsalmi M, Almarzouki H. Computer vision syndrome among health sciences students in Saudi Arabia: prevalence and risk factors. Cureus. 2020 Feb 20;12(2). DOI: [10.7759/cureus.7060](https://doi.org/10.7759/cureus.7060)
17. Cantó‐Sancho N, Sánchez‐Brau M, Ivorra‐Soler B, Seguí‐Crespo M. Computer vision syndrome prevalence according to individual and video display terminal exposure characteristics in Spanish university students. International journal of clinical practice. 2021 Mar;75(3):e13681. Doi: [10.1111/ijcp.13681](https://doi.org/10.1111/ijcp.13681)
18. Gammoh Y. Digital eye strain and its risk factors among a university student population in Jordan: a cross-sectional study. Cureus. 2021 Feb 26;13(2). doi: 10.7759/cureus.13575.
19. Noreen, K., Ali K., et al., Computer vision syndrome (CVS) and its associated risk factors among undergraduate medical students in midst of COVID-19. Pakistan J. Ophthalmol., 2021. 37(1). Doi: 10.36351/pjo.v37i1.1124
20. Nwankwo B, Mumueh KP, Olorukooba AA, Usman NO. Computer vision syndrome: prevalence and associated risk factors among undergraduates in a tertiary institution in north western Nigeria. Kanem Journal of Medical Sciences. 2021 Dec 1;15(1):19-26. DOI: 10.36020/kjms.2021.1501.003
21. Wang L, Wei X, Deng Y. Computer vision syndrome during SARS-CoV-2 outbreak in university students: a comparison between online courses and classroom lectures. Frontiers in public health. 2021 Jul 8;9:696036. doi: [10.3389/fpubh.2021.696036](https://doi.org/10.3389/fpubh.2021.696036)
22. Zenbaba D, Sahiledengle B, Bonsa M, Tekalegn Y, Azanaw J, Kumar Chattu V. Prevalence of Computer Vision Syndrome and Associated Factors among Instructors in Ethiopian Universities: A Web-Based Cross-Sectional Study. ScientificWorldJournal. 2021 Oct 5;2021:3384332. doi: 10.1155/2021/3384332.
23. Turkistani AN, Al-Romaih A, Alrayes MM, Al Ojan A, Al-Issawi W. Computer vision syndrome among Saudi population: An evaluation of prevalence and risk factors. J Family Med Prim Care. 2021 Jun;10(6):2313-2318. doi: 10.4103/jfmpc.jfmpc_2466_20.
24. Huyhua Gutiérrez, S.C., Meléndez Tuesta, J., Odar Rojas, C.E., Ruiz Cruz, D., & Tejada Muñoz, S. (2021). Computer Vision Syndrome and academic stress in nursing students during the COVID-19 lockdown. *Journal of the University of Zulia* , *12* (35), 572–583. https://doi.org/10.46925//rdluz.35.33
25. Iqbal M, Elzembely H, Elmassry A, Elgharieb M, Assaf A, Ibrahim O, Soliman A. Computer vision syndrome prevalence and ocular sequelae among medical students: a university-wide study on a marginalized visual security issue. The Open Ophthalmology Journal. 2021 Sep 22;15(1). DOI:[10.2174/1874364102115010156](http://dx.doi.org/10.2174/1874364102115010156)
26. Akowuah PK, Nti AN, Ankamah-Lomotey S, Frimpong AA, Fummey J, Boadi P, Osei-Poku K, Adjei-Anang J. Digital device use, computer vision syndrome, and sleep quality among an African undergraduate population. Advances in Public Health. 2021;2021(1):6611348. Doi:<https://doi.org/10.1155/2021/6611348>
27. Selvaraj S, Ganesan DK, Jain T. A study on single versus multiple symptoms of computer vision syndrome (CVS) among engineering students in Kancheepuram District, Tamil Nadu. International Journal of Current Research and Review. 2021 Mar;13(6):51-5. DOI: <http://dx.doi.org/10.31782/IJCRR.2021.13605>
28. Fernandez-Villacorta D, Soriano-Moreno AN, Galvez-Olortegui T, Agui-Santivañez N, Soriano-Moreno DR, Benites-Zapata VA. Computer visual syndrome in graduate students of a private university in Lima, Perú. Archivos de la Sociedad Española de Oftalmología (English Edition). 2021 Oct 1;96(10):515-20. DOI: [10.1016/j.oftal.2020.12.003](https://doi.org/10.1016/j.oftal.2020.12.003)
29. Iqbal M, Said O, Ibrahim O, Soliman A. Visual Sequelae of Computer Vision Syndrome: A Cross‐Sectional Case‐Control Study. Journal of ophthalmology. 2021;2021(1):6630286.DOI: [10.1155/2021/6630286](https://doi.org/10.1155/2021/6630286)
30. Wangsan K, Upaphong P, Assavanopakun P, Sapbamrer R, Sirikul W, Kitro A, Sirimaharaj N, Kuanprasert S, Saenpo M, Saetiao S, Khamphichai T. Self-Reported Computer Vision Syndrome among Thai University Students in Virtual Classrooms during the COVID-19 Pandemic: Prevalence and Associated Factors. Int J Environ Res Public Health. 2022 Mar 28;19(7):3996. doi: 10.3390/ijerph19073996.
31. Munsamy AJ, Naidoo S, Akoo T, Jumna S, Nair P, Zuma S, Blose S. A case study of digital eye strain in a university student population during the 2020 COVID-19 lockdown in South Africa: evidence of an emerging public health issue. J Public Health Afr. 2022 Oct 4;13(3):2103. doi: 10.4081/jphia.2022.2103.
32. Almousa AN, Aldofyan MZ, Kokandi BA, Alsubki HE, Alqahtani RS, Gikandi P, Alghaihb SG. The impact of the COVID-19 pandemic on the prevalence of computer vision syndrome among medical students in Riyadh, Saudi Arabia. International Ophthalmology. 2023 Apr;43(4):1275-83.
33. Wangsan K, Upaphong P, Assavanopakun P, Sapbamrer R, Sirikul W, Kitro A, Sirimaharaj N, Kuanprasert S, Saenpo M, Saetiao S, Khamphichai T. Self-reported computer vision syndrome among Thai university students in virtual classrooms during the COVID-19 pandemic: prevalence and associated factors. International journal of environmental research and public health. 2022 Mar 28;19(7):3996.
34. Coronel-Ocampos J, Gómez J, Gómez A, Quiroga-Castañeda PP, Valladares-Garrido MJ. Computer visual syndrome in medical students from a private university in Paraguay: a survey study. Frontiers in public health. 2022 Jul 14;10:935405.DOI: [10.3389/fpubh.2022.935405](https://doi.org/10.3389/fpubh.2022.935405)
35. Gebresellassie MG, Sisay H, Getahun B, Abera E, Birhanu H, Ashenafi T. Prevalence of computer vision syndrom and predisposing factors among engineering students in Hawass University institution of techenology campus, Hawassa, Ethiopia, 2019. The Open Ophthalmology Journal. 2022 Dec 20;16(1).[DOI: 10.2174/18743641-v16-e220128-2021-44](http://dx.doi.org/10.2174/18743641-v16-e220128-2021-44)
36. Imran M, Ahmad MS, Shaik RA, Alosaimi A, Almaymuni K, Albaradie N, Almotairi D, Alqahtani SM. Digital Eye Strain among undergraduate medical students at Majmaah University, Saudi Arabia: CROSS-SECTIONAL STUDY. Eur J Mol Clin Med. 2022;9(7):651À662.DOI: 10.1080/14790718.2022.2081330
37. Uwimana A, Ma C, Ma X. Concurrent rising of dry eye and eye strain symptoms among university students during the COVID-19 pandemic era: a cross-sectional study. Risk Management and Healthcare Policy. 2022 Jan 1:2311-22. DOI: [10.2147/RMHP.S388331](https://doi.org/10.2147/rmhp.s388331)
38. Lotfy NM, Shafik HM, Nassief M. Risk factor assessment of digital eye strain during the COVID-19 pandemic: a cross-sectional survey. Medical Hypothesis, Discovery and Innovation in Ophthalmology. 2022 Dec 3;11(3):119.<https://doi.org/10.51329/mehdiophthal1455>
39. Roy S, Sharif AB, Chowdhury S, Iktidar MA. Unavoidable online education due to COVID-19 and its association to computer vision syndrome: a cross-sectional survey. BMJ Open Ophth. 2022;7(1): e001118.doi:10.1136/ bmjophth-2022-001118
40. Lindo-Cano EF, García-Monge VA, Castillo-Cadillo KJ, Sánchez-Tirado EA, Távara IM, Morales J. Computer-digital vision syndrome among university students of Lima City. The Open Public Health Journal. 2022 Nov 8;15(1).[DOI: 10.2174/18749445-v15-e2208104](http://dx.doi.org/10.2174/18749445-v15-e2208104)
41. Estrada Araoz EG, Paricahua Peralta JN, Zuloaga Araoz MC, Gallegos Ramos NA, Paredes Valverde Y, Quispe Herrera R, Velásquez Giersch L. Prevalence of computer vision syndrome in Peruvian university students during the COVID-19 health emergency. AVFT [Internet]. 2023Apr.8 [cited 2025Apr.3];41(4). Doi: http://doi.org/10.5281/zenodo.6945062
42. AlDarrab A, Khojah AA, Al-Ghazwi MH, Al-Haqbani YJ, Al-Qahtani NM, Al-Ajmi MN, Alenezi SH, Almasoud MK, Al-Yahya AF. Magnitude and determinants of computer vision syndrome among college students at a Saudi university. Middle East African Journal of Ophthalmology. 2021 Oct 1;28(4):252-6. doi: 10.4103/meajo.meajo_272_21.
43. Gerena Pallares, L. C., Vargas Rodríguez, L. J., Niño Avendaño, C. A., Uyaban, G. C., & Ballesteros Virgen, Y. . (2022). Prevalence of computer visual syndrome in medical students from the city of Tunja during the pandemic. *Revista Colombiana De Salud Ocupacional*, *12*(1), e-7916. <https://doi.org/10.18041/2322-634X/rcso.1.2022.7916>
44. Vargas Rodríguez LJ, Espitia Lozano N, de la Peña Triana HM, Vargas Vargas JL, Mogollón Botía DM, Pobre Vinasco AM, Tristancho Rincón MA, Acosta Pérez CA, Sarria Carreño MC, Contento Suescun G. Computer visual syndrome in university students in times of pandemic. Arch Soc Esp Oftalmol (Engl Ed). 2023 Feb;98(2):72-77. doi: 10.1016/j.oftale.2022.08.009.
